# Effect of varenicline on major adverse liver outcomes in alcohol‐associated liver disease: An exploratory analysis

**DOI:** 10.1111/acer.70160

**Published:** 2025-09-15

**Authors:** Pojsakorn Danpanichkul, Yanfang Pang, Donghee Kim, Thanathip Suenghataiphorn, Donghyun Ko, Andrew F. Ibrahim, Vitchapong Prasitsumrit, Kwanjit Duangsonk, Mazen Noureddin, Karn Wijarnpreecha, Suthat Liangpunsakul

**Affiliations:** ^1^ Department of Internal Medicine Texas Tech University Health Sciences Center Lubbock Texas USA; ^2^ Affiliated Hospital of Youjiang Medical University for Nationalities Baise Guangxi China; ^3^ National Immunological Laboratory of Traditional Chinese Medicines Baise Guangxi China; ^4^ Center for Medical Laboratory Science Affiliated Hospital of Youjiang Medical University for Nationalities Baise Guangxi China; ^5^ Division of Gastroenterology and Hepatology Stanford University School of Medicine Stanford California USA; ^6^ Department of Internal Medicine Griffin Hospital Derby Connecticut USA; ^7^ Department of Internal Medicine, Bridgeport Hospital Yale New Haven Health Bridgeport Connecticut USA; ^8^ School of Medicine Texas Tech University Health Sciences Center Lubbock Texas USA; ^9^ Department of Microbiology, Faculty of Medicine Chiang Mai University Chiang Mai Thailand; ^10^ Houston Research Institute and Houston Methodist Hospital Houston Texas USA; ^11^ Division of Gastroenterology and Hepatology, Department of Medicine University of Arizona College of Medicine Phoenix Arizona USA; ^12^ Division of Gastroenterology and Hepatology, Department of Internal Medicine Banner University Medical Center Phoenix Arizona USA; ^13^ BIO5 Institute University of Arizona College of Medicine‐Phoenix Phoenix Arizona USA; ^14^ Division of Gastroenterology and Hepatology, Department of Medicine Indiana University School of Medicine Indianapolis Indiana USA; ^15^ Department of Biochemistry and Molecular Biology Indiana University School of Medicine Indianapolis Indiana USA

**Keywords:** addiction, alcohol‐related liver disease, liver disease, public health, substance use disorder

## Abstract

**Background:**

Varenicline, a partial agonist of the α4β2 nicotinic acetylcholine receptor, is effective for smoking cessation and has shown promise in treating alcohol use disorder (AUD). However, its impact on patients with concurrent alcohol‐associated liver disease (ALD) remains understudied. We aimed to evaluate the association between varenicline use and long‐term clinical outcomes in this population.

**Methods:**

We conducted a retrospective cohort study using the TriNetX federated network of deidentified electronic health records. Adults with diagnoses of both ALD and AUD were included. Patients prescribed varenicline were compared to those receiving FDA‐approved AUD pharmacotherapies (acamprosate or naltrexone), using 1:1 propensity score matching based on demographics, comorbidities, medications, and laboratory values. The primary outcome was major adverse liver outcomes (MALO); secondary outcomes included all‐cause mortality and other liver‐related complications. Hazard ratios (HRs) were estimated using Cox proportional hazards models over a five‐year follow‐up period.

**Results:**

A total of 1278 patients were included after matching. Varenicline use was associated with lower all‐cause mortality (14.4% vs. 17.4%; HR 0.75, 95% CI 0.57–0.99) and a significantly reduced risk of hepatic encephalopathy (3.5% vs. 6.7%; HR 0.47, 95% CI 0.28–0.79). Although overall MALO rates were similar between groups (17.3% vs. 17.6%; HR 0.89, 95% CI 0.66–1.20), subgroup analyses revealed reduced MALO incidence among females and all‐cause mortality among individuals aged ≥65 years.

**Conclusion:**

In this real‐world cohort study, varenicline use was associated with improved survival and a lower risk of hepatic encephalopathy compared to standard AUD pharmacotherapies in patients with co‐occurring ALD and AUD. These findings support further investigation of varenicline as a potential therapeutic option, ideally through randomized controlled trials.

## Graphical abstract

This real‐world study assessed whether varenicline improves outcomes in patients with ALD and AUD. Using data from a large electronic health record network, 1278 matched patients were analyzed. Varenicline use was associated with reduced all‐cause mortality and hepatic encephalopathy compared with FDA‐approved AUD therapies (acamprosate or naltrexone), though overall major liver outcomes were similar. Benefits were more pronounced in women and older adults. Findings suggest varenicline may be a promising alternative for select ALD patients, warranting further randomized trials.

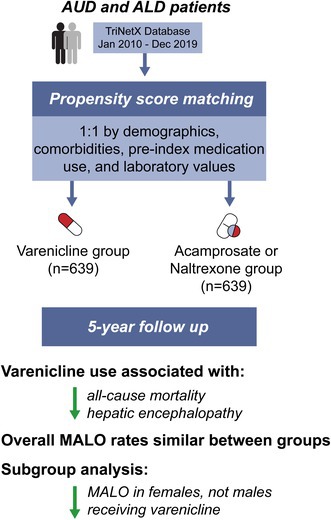

## INTRODUCTION

Alcohol is a major global cause of disease (Collaborators, 2018, 2025; Danpanichkul, Chen, et al., 2024). Hazardous drinking and alcohol use disorder (AUD) are distinct yet clinically significant consequences of alcohol consumption (Mellinger et al., 2023). Hazardous drinking increases the risk of acute and chronic alcohol‐related harm but does not necessarily indicate adverse health effects (MacKillop et al., 2022). In contrast, AUD is a psychiatric disorder characterized by impaired control over alcohol consumption and associated symptoms (Arab et al., 2025). While brief interventions may benefit, some individuals with AUD typically require more comprehensive therapies to achieve and sustain long‐term abstinence (Heilig et al., 2021; Mellinger et al., 2023). However, access to AUD treatment, including pharmacological and nonpharmacological therapies, remains limited. For instance, a US retrospective cohort study of veterans with cirrhosis due to alcohol‐associated liver disease (ALD) and AUD found that only 14% received any form of AUD treatment, with <1% receiving pharmacotherapy (Rogal et al., 2020). Across the spectrum of steatotic liver disease, ALD carries the greatest risk of liver‐related events, and these liver‐related events are driven by alcohol consumption (Kim, Ko, et al., 2025; Potts et al., 2013).

Varenicline, a partial agonist targeting the α4β2 nicotinic acetylcholine receptor subtype, is currently approved for smoking cessation (Ebbert et al., 2015; Guo et al., 2022). It exerts its effects by modulating nicotine‐induced receptor activity, thereby reducing cravings and alleviating withdrawal symptoms (Coe et al., 2005). Because of its action on the mesolimbic reward system, which is also implicated in AUD, varenicline has been investigated as a potential treatment for AUD (Diaz, Konig, et al., 2025). Several randomized controlled trials (RCTs) have explored its efficacy in promoting alcohol abstinence, with findings suggesting that varenicline may reduce alcohol cravings and consumption (Phimarn et al., 2023). Despite these encouraging results, critical knowledge gaps remain regarding varenicline's impact on long‐term clinical outcomes in individuals with AUD. In particular, its effects on mortality and major adverse liver outcomes (MALO) have not been systematically evaluated, even though numerous studies have examined its role in smoking cessation and short‐term alcohol reduction (Ebbert et al., 2015; Hurt et al., 2018). Moreover, prior RCTs of varenicline in AUD populations have largely excluded or overlooked patients with important medical comorbidities, particularly ALD, a condition that frequently coexists with AUD and significantly influences prognosis (Phimarn et al., 2023; Ramkissoon & Shah, 2022).

To address this critical evidence gap, we conducted a large‐scale, real‐world study using a federated electronic health record (EHR) database to examine the association between varenicline use and long‐term clinical outcomes in patients with both AUD and ALD. By focusing on a high‐risk, comorbidity‐laden population that is often underrepresented in RCTs, this study offers novel insights into the potential liver‐related and survival benefits of varenicline, extending beyond its established role in smoking cessation and alcohol use disorder treatment.

## METHODS

### Study design

We conducted our analysis using TriNetX (TriNetX, Inc., Cambridge, MA, USA), a global federated research platform that aggregates anonymized electronic medical records from 103 healthcare institutions worldwide (Ahn et al., 2025; Danpanichkul, Kim, Nah, et al., 2025). This platform enables large‐scale observational studies without requiring prior institutional review board approval. Data for this study were retrieved from the database on July 20, 2025. Eligible participants included adults aged ≥18 years diagnosed with ALD from January 2010 to December 2019 at any fibrosis stage who also had a concurrent diagnosis of AUD. Patients were identified using the International Classification of Diseases (ICD) codes (Appendix S1). To ensure a homogeneous study population, we excluded individuals with other liver diseases such as viral hepatitis, autoimmune liver disease, and hemochromatosis, as well as those with a history of liver transplantation or pregnancy at baseline (Appendix S2).

To compare treatment outcomes, we created two cohorts of patients with ALD and AUD. Cohort A comprised individuals prescribed varenicline, excluding those receiving acamprosate or naltrexone, while Cohort B included those prescribed acamprosate or naltrexone without concurrent use of varenicline. We selected varenicline as a comparator in this study based on its unique pharmacological mechanism and established therapeutic role. Unlike conventional agents such as naltrexone or acamprosate, varenicline targets the α4β2 nicotinic acetylcholine receptor, modulating dopaminergic pathways implicated in both alcohol and nicotine dependence (Bordia et al., 2012; Yang et al., 2022). This dual mechanism is particularly relevant given the high prevalence of smoking among individuals with AUD, positioning varenicline as a potentially effective option for simultaneously addressing both conditions (Beard et al., 2017; Falk et al., 2006; Witvorapong & Vichitkunakorn, 2021). Furthermore, emerging evidence suggests that varenicline may reduce alcohol craving and consumption (Litten et al., 2013).

This study employed an active‐comparator design within the TriNetX platform, ensuring a direct comparison between varenicline and Food and Drug Administration (FDA)‐approved AUD medications (acamprosate and naltrexone). Exposure to varenicline or comparator drugs (acamprosate or naltrexone) was defined by the first recorded prescription event in the electronic health record, as captured and mapped within the TriNetX platform. Consistent with an intention‐to‐treat (ITT) analytical framework commonly applied in real‐world evidence studies (de Cates et al., 2024), a single prescription order was considered sufficient to define exposure. TriNetX does not perform imputation for missing data; instead, a complete‐case analysis was employed (Hung et al., 2025).

To minimize confounding, we applied propensity score matching (PSM) using logistic regression to balance baseline characteristics between cohorts. A 1:1 greedy nearest‐neighbor matching algorithm with a caliper width of 0.1 pooled standard deviations was utilized. Matching variables included demographic factors (age, sex, race/ethnicity); clinical characteristics such as substance use and psychiatric disorders, metabolic and liver‐related conditions (e.g., cirrhosis, hepatic failure, diabetes, and obesity), cardiovascular and kidney diseases, and lipid disorders. Procedures related to behavioral health, substance use treatment, and Transjugular Intrahepatic Portosystemic Shunt were considered. Medications included cardiovascular, metabolic, and neuropsychiatric agents. Laboratory values encompassed liver function, coagulation, renal function, glucose, lipids, and blood counts (Appendix S2). A standardized mean difference (SMD) of 0.1 or less between groups is generally considered indicative of negligible imbalance. Patients were followed for up to 5 years to assess clinical outcomes. The primary endpoint was a composite measure of MALO, encompassing ascites, hepatic encephalopathy, variceal bleeding, spontaneous bacterial peritonitis (SBP), portal hypertension, hepatorenal syndrome, and hepatopulmonary syndrome. The secondary outcome was all‐cause mortality. Time‐to‐event analysis was performed using univariate survival analysis with interval probability estimates. Patients were censored upon experiencing an outcome or at predefined censoring events. Subgroup analyses by sex, age, and smoking status were conducted to evaluate potential effect modification. These factors are clinically relevant, as the global burden of ALD is rising among women and remains high in older adults (Danpanichkul, Pang, Mahendru, et al., 2025; Danpanichkul, Suparan, et al., 2024). Moreover, sex, age, and smoking status may influence both disease trajectory and treatment response (Kezer et al., 2021; Tonstad et al., 2020). Evaluating these subgroups enhances the clinical relevance and generalizability of our findings.

### Statistical analyses

All analyses were conducted using the TriNetX platform. Baseline characteristics were summarized as mean ± standard deviation (SD) for continuous variables and as counts (percentages) for categorical variables. Propensity score matching (PSM) was performed using logistic regression to achieve covariate balance between groups. Kaplan–Meier analysis was used to estimate the probability of outcomes over time, with event probabilities calculated at each daily interval. To account for patients who exited the cohort during the follow‐up period, right censoring was applied. Specifically, patients were censored at the date of their last recorded clinical fact in the TriNetX network, which could include a diagnosis, procedure, lab result, medication administration, or clinical encounter

Kaplan–Meier plots were used to estimate outcomes over a 5‐year period from the index date. To enhance baseline accuracy and address potential delays in ICD code documentation, the outcome was defined by events recorded after the first 3 months to 5 years after the index event. This approach helped capture preexisting conditions that may have been recorded later in the EHR and minimize the risk of immortal time bias. Patients were censored at the time of the outcome, death, or loss to follow‐up, whichever came first. Individuals with documented evidence of the outcome prior to the index date (e.g., prior ascites or hepatic encephalopathy) were excluded from the respective time‐to‐event analyses. Cox proportional hazards models were used to estimate hazard ratios (HRs) and 95% confidence intervals (CIs) for associations between exposure and clinical outcomes, focusing on the first occurrence of each outcome. The proportional hazards assumption was evaluated using the generalized Schoenfeld residual method implemented within the TriNetX platform. The analysis was limited to time‐to‐first event, consistent with the Kaplan–Meier methodology (Wang et al., 2022).

## RESULTS

### Baseline characteristics before and after propensity score matching

**FIGURE 1:**
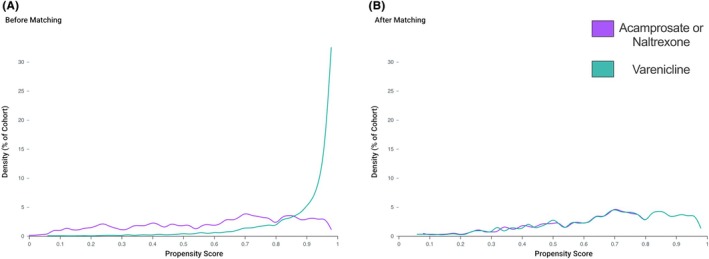
Propensity Score of cohorts prescribed varenicline versus acamprosate or naltrexone.

The baseline characteristics are mostly imbalanced between the two cohorts (Appendix S3 and Figure 1). After propensity score matching, a total of 1278 individuals with ALD were included in the final analysis, with 639 patients in both the varenicline and FDA‐approved AUD pharmacotherapy groups (acamprosate or naltrexone) (Table 1). The two cohorts were mostly well‐balanced across demographic, diagnostic, medication, and laboratory variables (Table 1 and Figure 1). The mean age was 53.3 ± 9.6 years in the varenicline group and 53.2 ± 10.7 years in the comparator group. Female patients comprised 30.8% of the varenicline group and 34.1% of the acamprosate/naltrexone group. Racial and ethnic compositions were similar across groups, with White patients representing over 74% in both cohorts and comparable representation of Black, Asian, and Hispanic individuals (Table 1).

**TABLE 1:** Baseline patient demographics and characteristics after propensity score matching.

Characteristic	Varenicline (*N* = 639)	Acamprosate or naltrexone (*N* = 639)	Std diff.
Demographic			
Age at index (years)	53.3 ± 9.6	53.2 ± 10.7	0.014
Female (%)	197 (30.8)	218 (34.1)	0.070
White (%)	486 (76.1)	474 (74.2)	0.043
Not Hispanic or Latino (%)	477 (74.6)	463 (72.5)	0.050
Black or African American (%)	79 (12.4)	88 (13.8)	0.042
Asian (%)	13 (2.0)	13 (2.0)	<0.001
Comorbidities			
Smoking	200 (31.3)	199 (31.1)	0.003
Mental/behavioral disorders (%)	538 (84.2)	566 (88.6)	0.128
Nicotine dependence (%)	421 (65.9)	422 (66.0)	0.003
Opioid‐related disorders (%)	37 (5.8)	40 (6.3)	0.020
Mood disorders (%)	256 (40.1)	267 (41.8)	0.035
Type 2 diabetes mellitus (%)	121 (18.9)	122 (19.1)	0.004
Obesity (%)	115 (18.0)	118 (18.5)	0.012
Medications			
CNS medications (%)	545 (85.3)	537 (84.0)	0.035
Opioid analgesics (%)	419 (65.6)	414 (64.8)	0.016
Antidepressants (%)	341 (53.4)	345 (54.0)	0.013
Laboratory values			
Sodium (mmol/L)	137.4 ± 3.9	137.6 ± 4.0	0.063
Creatinine (mg/dL)	0.9 ± 0.6	0.8 ± 0.4	0.177
Total bilirubin (mg/dL)	1.0 ± 1.7	1.4 ± 2.8	0.193
Albumin (g/dL)	3.9 ± 0.7	3.8 ± 0.7	0.213
HbA1c (%)	5.8 ± 1.5	5.8 ± 1.6	0.009
Others			
Smoking cessation counseling visit	62 (9.7%)	63 (9.9%)	0.005

Abbreviations: CNS, central nervous system; HbA1c, glycated hemoglobin; Std diff, standardized mean differences.

Key comorbidities, including mood disorders, nicotine dependence, smoking status, type 2 diabetes mellitus, and obesity, were comparable between groups. Notably, both groups had high rates of psychiatric diagnoses (84.2% vs. 88.6%) and central nervous system (CNS) medication use (85.3% vs. 84.0%). Use of analgesics, opioid analgesics, and antidepressants was nearly identical. Laboratory parameters such as sodium and glycated hemoglobin showed good balance between cohorts (Table 1 and Figure 1). However, total bilirubin, creatinine, albumin, and mental/behavioral disorders demonstrated residual imbalances despite propensity score matching (standardized mean differences ≥0.10) (Table 1).

### Association of varenicline use with clinical outcomes in ALD

**FIGURE 2:**
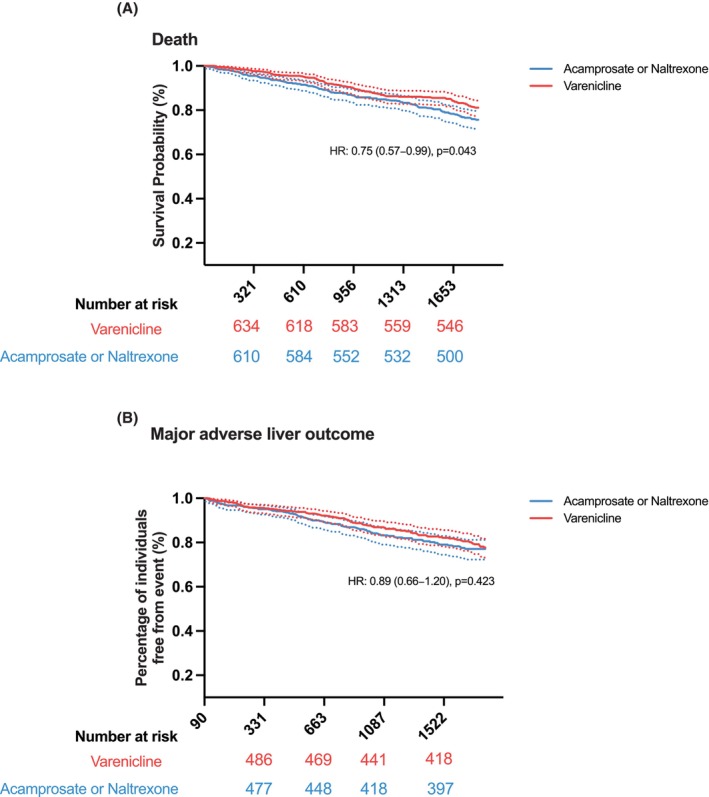
(A) Survival probability of patients in cohorts prescribed varenicline versus acamprosate or naltrexone. (B) Kaplan–Meier curves of major adverse liver outcomes.

In this matched analysis, varenicline use was associated with a lower risk of all‐cause mortality compared to acamprosate or naltrexone (14.4% vs. 17.4%; HR: 0.75, 95% CI: 0.57–0.99) (Table 2 and Figure 2A).

**TABLE 2:** Clinical outcome of cohorts.

Outcome	Varenicline events, *N* (%)	Acamprosate or naltrexone events, *N* (%)	Hazard ratio (95% CI)	*p*‐Value
Death	91 (14.4%)	108 (17.4%)	0.75 (0.57–0.99)	**0.043**
Liver‐related outcomes				
Major adverse liver outcome^a^	86 (17.3%)	86 (17.6%)	0.89 (0.66–1.20)	0.429
Portal hypertension	63 (11.7%)	74 (13.9%)	0.76 (0.55–1.07)	0.113
Hepatic encephalopathy	22 (3.5%)	41 (6.7%)	0.47 (0.28–0.79)	**0.003**
Ascites	74 (13.7%)	67 (12.4%)	1.00 (0.72–1.39)	0.980
Spontaneous bacterial peritonitis	16 (2.5%)	16 (2.6%)	0.91 (0.45–1.82)	0.785
Variceal bleeding	10 (1.6%)	14 (2.2%)	0.66 (0.29–1.48)	0.304
Hepatorenal syndrome	19 (3.1%)	18 (2.9%)	0.97 (0.51–1.84)	0.914
Hepatopulmonary syndrome	10 (1.6%)	10 (1.6%)	0.90 (0.13–6.36)	0.912

Note: Bold refer to significance value.

Abbreviations: CI, confidence interval; HR, hazard ratio.

^a^ Major adverse liver outcomes are calculated by combining ascites, hepatic encephalopathy, variceal bleeding, hepatorenal syndrome, hepatopulmonary syndrome, and spontaneous bacterial peritonitis.

Regarding liver‐related outcomes, the incidence of MALO was comparable between the two groups (17.3% vs. 17.6%; HR: 0.89, 95% CI: 0.66–1.20) (Table 2 and Figure 2B). Among individual liver complications, the varenicline group had a notably lower incidence of hepatic encephalopathy (3.5% vs. 6.7%; HR: 0.47, 95% CI: 0.28–0.79). No significant differences were observed in the rates of ascites (13.7% vs. 12.4%; HR: 1.00, 95% CI: 0.72–1.39), portal hypertension (11.7% vs. 13.9%; HR: 0.76, 95% CI: 0.55–1.07), spontaneous bacterial peritonitis (2.5% vs. 2.6%; HR: 0.91, 95% CI: 0.45–1.82), variceal bleeding (1.6% vs. 2.2%; HR: 0.66, 95% CI: 0.29–1.48), hepatorenal syndrome (3.1% vs. 2.9%; HR: 0.97, 95% CI: 0.51–1.84), or hepatopulmonary syndrome (1.6% vs. 1.6%; HR: 0.90, 95% CI: 0.13–6.36) (Table 2).

### Subgroup analyses

Subgroup analyses were conducted by age group, sex, and smoking status (Table 3). Among patients aged ≥65 years, varenicline was associated with a trend toward, yet nonsignificant, a lower risk of MALO (15.3% vs. 21.3%; HR: 0.58, 95% CI: 0.33–1.02) and a significantly lower risk of all‐cause mortality (12.6% vs. 19.8%; HR: 0.57, 95% CI: 0.34–0.98) compared to FDA‐approved AUD pharmacotherapies. Female patients receiving varenicline also experienced a significantly lower risk of MALO compared to the comparator group (14.0% vs. 19.7%; HR: 0.56, 95% CI: 0.31–1.00), while mortality differences were not statistically significant. In contrast, outcomes among male patients were comparable between groups (Table 3). No significant differences in MALO or mortality were observed based on smoking status. The HRs for MALO in smokers and nonsmokers were 1.00 (95% CI: 0.64–1.54) and 0.83 (95% CI: 0.53–1.32), respectively. Similarly, mortality risk did not differ significantly between treatment groups within either smoking subgroup (Table 3).

**TABLE 3:** Clinical outcome of cohorts by subgroup.

	Major adverse liver outcome^a^				Death			
	Varenicline event (%)	Acamprosate or naltrexone event (%)	HR (95% CI)	*p*	Varenicline event (%)	Acamprosate or naltrexone event (%)	HR (95% CI)	*p*
By age group								
18–64 years	54 (16.77)	50 (16.29)	0.94 (0.64 to 1.39)	0.770	61 (14.81)	58 (14.25)	0.92 (0.64 to 1.32)	0.642
≥65 years	21 (15.33)	30 (21.28)	0.58 (0.33 to 1.02)	0.055	22 (12.64)	34 (19.77)	0.57 (0.34 to 0.98)	**0.039**
By sex								
Female	19 (13.97)	27 (19.71)	0.56 (0.31 to 1.00)	**0.047**	25 (14.04)	32 (18.29)	0.67 (0.39 to 1.12)	0.124
Male	62 (18.08)	53 (15.32)	1.04 (0.72 to 1.50)	0.833	68 (16.04)	73 (17.38)	0.80 (0.60 to 1.20)	0.193
Smoking status								
Smoker	40 (17.17)	40 (17.17)	1.00 (0.64 to 1.54)	0.982	42 (14.05)	52 (17.75)	0.77 (0.51 to 1.16)	0.212
Nonsmoker	35 (16.59)	37 (16.97)	0.83 (0.53 to 1.32)	0.441	48 (17.65)	43 (15.81)	0.99 (0.66 to 1.49)	0.961

Note: Bold refer to significance value.

Abbreviation: CI, confidence interval; HR, hazard ratio.

^a^ Major adverse liver outcomes are calculated by combining ascites, hepatic encephalopathy, variceal bleeding, hepatorenal syndrome, hepatopulmonary syndrome, and spontaneous bacterial peritonitis.

## DISCUSSION

The majority of patients with ALD do not receive appropriate treatment for AUD despite the well‐established link between alcohol consumption and liver disease progression (Haque & Leggio, 2024; Rogal et al., 2020; Vannier et al., 2022). Addressing this significant treatment gap requires a comprehensive and multifaceted approach. Efforts should include integrating addiction education into medical and healthcare training curricula, equipping clinicians with the necessary skills to identify and manage AUD effectively (Haque & Leggio, 2024). Acamprosate and naltrexone are FDA‐approved medications for the treatment of AUD. Varenicline, though not an FDA‐approved treatment for AUD, has been considered a promising option, particularly for individuals who smoke and are unable to use FDA‐approved AUD medications (Haque & Leggio, 2024). To our knowledge, this is the first study to directly compare varenicline with FDA‐approved AUD pharmacotherapies. This real‐world data study evaluates mortality and MALO in patients with concurrent ALD and AUD who were prescribed varenicline compared to those receiving acamprosate or naltrexone. Our findings suggest that varenicline is associated with a significant reduction in both overall mortality and hepatic encephalopathy over a 5‐year follow‐up period. Given that both FDA‐approved AUD pharmacotherapies and varenicline have demonstrated efficacy in reducing alcohol consumption, one might assume that their effects on MALO and mortality would be similar (Hofer et al., 2023). However, our study found that varenicline, a medication primarily used for smoking cessation, was associated with a lower risk of mortality and hepatic encephalopathy compared to FDA‐approved AUD medications in patients with ALD and AUD.

Varenicline demonstrated heterogeneous effects on different subcomponents of MALOs, likely reflecting its targeted efficacy in hepatic encephalopathy, a neuroinflammatory condition (Mizrachi et al., 2021). By acting on nicotinic receptors, varenicline may reduce neuroinflammation and ammonia‐related toxicity, thereby improving symptoms of hepatic encephalopathy. In contrast, outcomes such as ascites and variceal bleeding are primarily driven by portal hypertension, a hemodynamic complication that varenicline does not directly affect, highlighting that its benefits are predominantly neuro‐modulatory rather than indicative of broad improvement in the underlying liver pathology (Tonon & Piano, 2023). It is important to note that although rates of hepatic encephalopathy differed significantly between the two cohorts, the overall composite MALO outcome remained statistically similar. Therefore, interpretation of varenicline's effect on MALO should be approached with caution, as most individual components of the composite outcome did not reach statistical significance. This suggests that the observed association may be driven by specific outcomes rather than reflecting a consistent effect across all liver‐related events.

Several potential explanations may account for this finding. One is the combined effect of alcohol and smoking cessation, as prior cohort studies have suggested that alcohol consumption often decreases during smoking cessation attempts, which could contribute to improved liver‐related outcomes (Vinci et al., 2024). Another possible explanation is that patients receiving FDA‐approved pharmacotherapy for AUD may have had greater AUD severity at baseline, potentially leading to worse outcomes. However, the TriNetX database did not include detailed data on AUD severity, limiting our ability to assess this factor (Kranzler & Soyka, 2018). Further studies are warranted to better understand the underlying mechanisms driving these differences and to explore the potential role of varenicline as an alternative treatment option for patients with AUD and ALD. This highlights that our findings do not challenge the efficacy of acamprosate or naltrexone but rather suggest that varenicline may serve as an effective alternative or adjunct to FDA‐approved AUD pharmacotherapies. Notably, a prior rat model study demonstrated that the combination of varenicline and naltrexone resulted in greater reductions in alcohol consumption than either medication alone (de Bejczy et al., 2015).

Subgroup analyses revealed that female patients with ALD treated with varenicline had lower rates of MALO compared to those receiving acamprosate or naltrexone, whereas no significant differences were observed in male patients. While prior studies have reported minimal sex differences with FDA‐approved AUD therapies (McKee & McRae‐Clark, 2022), and some evidence indicates a greater reduction in alcohol consumption among females treated with varenicline (O'Malley et al., 2018), our findings suggest that varenicline may confer additional liver‐related benefits in women. This effect may be attributable to women's greater susceptibility to alcohol‐induced liver injury and consequently larger benefits from reduced alcohol intake (Lee et al., 2012). Older adults also appeared to derive greater benefit, consistent with previous studies demonstrating enhanced alcohol reduction with varenicline in this population (Falk et al., 2015). No significant differences in mortality or MALO were observed based on smoking status, aligning with clinical trials indicating varenicline's efficacy is independent of smoking behavior (Litten et al., 2013). It is important to note that these subgroup findings are exploratory and limited by relatively small sample sizes (Serdar et al., 2021).

Our study benefits from a large sample size, real‐world clinical data, and the use of rigorous matching methods to approximate a trial‐like comparison. These strengths enhance the generalizability of our findings and help mitigate some biases inherent to observational studies. However, several limitations should be considered. First, using electronic health records from an administrative database introduces potential concerns about data completeness (Haut et al., 2012). Certain clinical details may be undocumented by healthcare providers or stored in free‐text format, making them inaccessible for structured analysis (Anson et al., 2024). This could lead to missing information on key variables that influence treatment outcomes. Second, the absence of detailed data on dosage, adherence, and dose escalation for AUD pharmacotherapies restricts our ability to evaluate potential dose‐dependent effects on alcohol consumption and liver‐related outcomes (Anton, 2008). Variability in medication adherence and treatment intensity may have influenced our results but could not be accounted for due to these data limitations. To address the lack of data on dosage and adherence, future studies should consider linking EHR data with pharmacy claims or incorporating proxy measures such as prescription persistence or follow‐up encounters. Third, the absence of detailed information on alcohol consumption patterns, potential unrecorded alcohol use, and undocumented liver events limits our ability to accurately classify patients (Danpanichkul, Suparan, Prasitsumrit, et al., 2025; Getzen et al., 2023). This limitation increases the risk of misclassification, especially in distinguishing ALD from metabolic dysfunction‐associated liver disease (MASLD) or metabolic dysfunction and alcohol‐associated liver disease (MetALD), conditions that share overlapping clinical features (Danpanichkul, Suparan, Diaz, et al., 2025; Diaz, Ajmera, et al., 2025; Kim, Danpanichkul, et al., 2025). Fourth, although PSM was applied, residual confounding remains. Key clinical variables such as creatinine, total bilirubin, degree of fibrosis, Child–Pugh score, and MELD score, critical indicators of liver disease severity, could not be matched (Kim et al., 2021; Peng et al., 2016; Zuluaga et al., 2024). It is possible that varenicline users had less severe baseline liver disease, as objective severity measures, including MELD, Child–Pugh, and AUDIT scores, were unavailable for matching. This may partly explain the higher rates of death and hepatic encephalopathy observed in the FDA‐approved AUD pharmacotherapy group. The inability to adjust for these factors could have influenced our findings. Additionally, calculation of the E‐value was not feasible due to the inherent limitations of the TriNetX database (VanderWeele & Ding, 2017). Lastly, a key limitation of our study is the lack of data on the duration of varenicline treatment. Without information on treatment length, we cannot determine whether sustained varenicline use confers greater benefits or whether early treatment discontinuation affects outcomes. Future research should investigate the impact of treatment duration to better elucidate varenicline's role in managing AUD and ALD.

In conclusion, this study provides preliminary evidence that varenicline may offer significant clinical benefits for patients with ALD and AUD. Varenicline use was associated with a lower risk of death and hepatic encephalopathy compared to FDA‐approved AUD pharmacotherapies. Given its dual efficacy in promoting smoking cessation and reducing alcohol consumption, varenicline may be a promising therapeutic option, particularly for patients who smoke or are unable to tolerate or access standard AUD medications (Diaz, Konig, et al., 2025; Leggio & Lee, 2017). However, due to the observational and exploratory nature of this analysis, the findings should be considered hypothesis generating. Rigorous randomized controlled trials are needed to confirm the safety and efficacy of varenicline in ALD, especially regarding liver‐related outcomes and its role in clinical practice.

## AUTHOR CONTRIBUTIONS

Conceptualization—Pojsakorn Danpanichkul. Data curation—Pojsakorn Danpanichkul, Andrew F. Ibrahim, Mazen Noureddin. Formal analysis—Pojsakorn Danpanichkul, Andrew F. Ibrahim, Kwanjit Duangsonk. Funding acquisition—No funding required. Investigation—Pojsakorn Danpanichkul, Yanfang Pang, Donghyun Ko. Methodology—Pojsakorn Danpanichkul, Andrew F. Ibrahim, Thanathip Suenghataiphorn. Project Administration—Pojsakorn Danpanichkul. Supervision—Mazen Noureddin. Validation—Pojsakorn Danpanichkul, Andrew F. Ibrahim, Yanfang Pang, Vitchapong Prasitsumrit. Visualization—Pojsakorn Danpanichkul, Vitchapong Prasitsumrit. Writing, original draft—Pojsakorn Danpanichkul, Thanathip Suenghataiphorn, Yanfang Pang. Writing, review, and editing—Karn Wijarnpreecha, Kwanjit Duangsonk, Donghee Kim, Suthat Liangpunsakul, Mazen Noureddin. All authors have read and approved the final version of the manuscript for submission.

## FUNDING INFORMATION

This study did not receive any funding.

## CONFLICT OF INTEREST STATEMENT

Suthat Liangpunsakul has served as a consultant for Durect. Mazen Noureddin has been on the advisory board for 89BIO, Gilead, Intercept, Pfizer, Novo Nordisk, Blade, Echosens, Fractyl, Terns, Siemens, and Roche diagnostics; has received research support from Allergan, BMS, Gilead, Galmed, Galectin, Genfit, Conatus, Enanta, Madrigal, Novartis, Pfizer, Shire, Viking, and Zydus; and is a minor shareholder or has stocks in Anaetos, Rivus Pharma, and Viking.

## INSTITUTIONAL REVIEW BOARD APPROVAL

The data utilized in this article were obtained from the publicly available TrinetX de‐identified database and, thus, did not necessitate any institutional review board approval, ethics clearance, or consent from study subjects.

## Supporting information


Appendices S1–S3

## Data Availability

The data that support the findings of this study are available from the TriNetX Analytics Network. https://trinetx.com.
