# Supplementary material for: Effect of varenicline on major adverse liver outcomes in alcohol‐associated liver disease: An exploratory analysis
Source: Alcohol Clin Exp Res (Hoboken). 2025 Sep 15;49(11):2451–60. doi: 10.1111/acer.70160 (PMC12638274; doi:10.1111/acer.70160)
Supplement: Supplementary file 1 — Appendices S1–S3 [file ACER-49-2451-s001.docx]

**Appendix S1.** Inclusion and Exclusion Criteria of Cohort

| **Domain** | **Cohort A: Alcohol-associated liver disease with Varenicline Use** | **Cohort B: Alcohol-associated liver disease with Acamprosate or Naltrexone Use** |
| --- | --- | --- |
| **Inclusion Criteria** | - Age ≥ 18 years  - Alcohol-associated liver disease (ICD-10 K70.x)  - Alcohol use disorder (ICD-10 F10)  - Prescribed Varenicline (RxNorm: 591622) on or after Jan 1, 2010  - No prescription for naltrexone or acamprosate | - Age ≥ 18 years  - Alcohol-associated liver disease (ICD-10 K70.x)  - Alcohol use disorder (ICD-10 F10)  - Prescribed acamprosate or naltrexone on or after Jan 1, 2010  - No Varenicline use (RxNorm: 591622) |
| **Exclusion Criteria** | - Other liver diseases (e.g., toxic, viral, autoimmune, genetic, cholestatic) (ICD-10 K71, B16–B19, K74, K75.4, K83.01, E83.01, E83.110, E88.01)  - Liver transplant status or procedure (ICD-10 Z94.4, SNOMED 18027006, CPT 47135, etc.)  - Malignancies  (ICD-10 C15–C96)  - Pregnancy or lactation  (ICD-10 O00–O9A, Z32.01, Z34.x, O92.x)  - All date constraints: Jan 1, 2010 – Dec 31, 2019 | Same as Cohort A |

**Appendix S2. Covariates Included in the Propensity Score Model**

**Demographics:**

Matching included sex (Male, Female), age at index, race/ethnicity (White, Black or African American, Asian, American Indian or Alaska Native, Native Hawaiian or Other Pacific Islander), and Hispanic or Latino ethnicity.

**Diagnoses:**

Patients were matched on a comprehensive set of clinical conditions, including substance use disorders (nicotine, cannabis, opioids, cocaine, sedatives, stimulants, inhalants, hallucinogens, and other psychoactive substances), psychiatric disorders (mood, anxiety, stress-related, dissociative, and somatoform disorders), metabolic and liver-related conditions (fibrosis, cirrhosis, hepatic failure, type 2 diabetes, malnutrition, fluid and electrolyte imbalances, magnesium disorders), cardiovascular diseases (hypertension, ischemic heart disease, other heart diseases, venous and lymphatic disorders), lipid disorders (hyperlipidemia, hyperglyceridemia, lipoprotein metabolism disorders), chronic kidney disease, and obesity-related conditions.

**Procedures:**

Matching included documentation of behavioral health interventions and substance use assessments, such as structured screenings (e.g., AUDIT, DAST) with brief or extended counseling sessions, enrollment in alcohol, smoking, or drug treatment programs, and interventional procedures like transjugular intrahepatic portosystemic shunt (TIPS).

**Medications:**

Patients were matched on a wide array of medication classes, including:

- Gastrointestinal, respiratory, cardiovascular, genitourinary, and central nervous system agents
- Vitamins, minerals, antimicrobials, immunological agents, and herbal therapies
- Specific cardiovascular drugs (e.g., lisinopril, furosemide, spironolactone, losartan, valsartan, hydralazine)
- Antihyperglycemic agents include insulin, GLP-1 receptor agonists, SGLT2 inhibitors, sulfonylureas (glipizide, glimepiride), and DPP-4 inhibitors (sitagliptin)
- Lipid-lowering agents, rifaximin, and a broad range of analgesics, antipsychotics, antidepressants, and sedatives

**Laboratory Values:**

Matching also accounted for laboratory measurements including electrolytes, renal function markers, glucose, complete blood counts, liver function tests (e.g., bilirubin, albumin), coagulation parameters (PT, INR, aPTT), lipid panels (HDL, LDL, total, non-HDL cholesterol, triglycerides), HbA1c, urate levels, blood pressure (systolic and diastolic), platelet indices, and protein or albumin levels in blood and urine.

**Appendix S3. Baseline patient demographics and characteristics before propensity score matching**

| Characteristic | Varenicline  (N=774) | Acamprosate or Naltrexone  (N=4,730) | Std diff. |
| --- | --- | --- | --- |
| Demographic |  |  |  |
| Age at Index (years) | 54.1 ± 9.6 | 48.8 ± 11.7 | 0.494 |
| Female (%) | 230 (29.7) | 1,449 (30.6) | 0.020 |
| White (%) | 588 (76.0) | 3,430 (72.5) | 0.079 |
| Not Hispanic or Latino (%) | 581 (75.1) | 3,347 (70.8) | 0.097 |
| Black or African American (%) | 100 (12.9) | 513 (10.8) | 0.064 |
| Asian (%) | 15 (1.9) | 89 (1.9) | 0.004 |
| **Comorbidities** |  |  |  |
| Smoking | 268 (34.6) | 836 (17.7) | 0.393 |
| Mental/Behavioral Disorders (%) | 671 (86.7) | 4,454 (94.2) | 0.256 |
| Nicotine Dependence (%) | 549 (70.9) | 2,175 (46.0) | 0.523 |
| Opioid Related Disorders (%) | 44 (5.7) | 368 (7.8) | 0.084 |
| Anxiety Disorders (%) | 258 (33.3) | 2,282 (48.2) | 0.307 |
| Mood Disorders (%) | 290 (37.5) | 2,583 (54.6) | 0.349 |
| Type 2 Diabetes Mellitus (%) | 151 (19.5) | 807 (17.1) | 0.063 |
| Obesity (%) | 143 (18.5) | 843 (17.8) | 0.017 |
| **Medications** |  |  |  |
| CNS Medications (%) | 669 (86.4) | 4,395 (92.9) | 0.214 |
| Opioid Analgesics (%) | 528 (68.2) | 3,221 (68.1) | 0.003 |
| Antidepressants (%) | 405 (52.3) | 2,839 (60.0) | 0.156 |
| **Laboratory values** |  |  |  |
| Sodium (mmol/L) | 137.4 ± 3.9 | 137.4 ± 4.2 | 0.015 |
| Creatinine (mg/dL) | 0.9 ± 0.5 | 0.8 ± 0.4 | 0.258 |
| Total bilirubin (mg/dL) | 0.9 ± 1.5 | 2.1 ± 3.7 | 0.409 |
| Albumin (g/dL) | 3.9 ± 0.7 | 3.6 ± 0.8 | 0.447 |
| HbA1c (%) | 5.9 ± 1.6 | 5.8 ± 1.6 | 0.093 |
| **Others** |  |  |  |
| Smoking cessation counselling visit | 76 (9.8) | 209 (4.4) | 0.211 |
